# Failure Analysis of Hat-Stringer-Stiffened Aircraft Composite Panels under Four-Point Bending Loading

**DOI:** 10.3390/ma15072430

**Published:** 2022-03-25

**Authors:** Binkai Li, Yu Gong, Yukui Gao, Mengqing Hou, Lei Li

**Affiliations:** 1 School of Aerospace Engineering and Applied Mechanics, Tongji University, Zhangwu Road 100#, Shanghai 200092, China; 1810398@tongji.edu.cn; 2 College of Aerospace Engineering, Chongqing University, Chongqing 400044, China; 3 School of Materials Science and Engineering, Tongji University, Caoan Road 4800#, Shanghai 201804, China; 12088@tongji.edu.cn; 4 Shanghai Key Laboratory of R&D for Metallic Function Materials, Tongji University, Caoan Road 4800#, Shanghai 201804, China; 5 Chinese Aeronautical Establishment, Xiaoguandongli 14#, Beijing 100029, China; houmq001@avic.com; 6 Shanghai Aircraft Design and Research Institute, Jinke Road 5188#, Shanghai 201210, China; lilei1@comac.cc

**Keywords:** composite laminate, hat-stringer-stiffened, four-point bending, failure mode

## Abstract

Hat-stringer-stiffened composite panels have been widely used in aircrafts. Accurate failure analysis of them is important for the safety and integrity of the fuselage. During the service period, these panels will bear not only the lateral force caused by the bending of fuselage, but also the radial pressure caused by the internal and external differential pressure during the take-off and landing of the aircraft. However, the latter case lacks investigation. Therefore, experimental and numerical studies for the static and fatigue failure of hat-stringer-stiffened composite panels under four-point bending loading have been performed in this work. To accurately predict the fatigue failure, a novel theoretical model has been proposed based on the fatigue damage theory. In addition, a user-defined subroutine USDFLD is developed for the implementation of the proposed theoretical model in Abaqus. Experimental results show that the main failure modes are the delamination of the skin and debonding between the girder flange and the skin. The experimental average value of the initial debonding load and displacement in static tests are 897.3 N and 10.8 mm, respectively. Predictions exhibit good agreement with experimental results with relative errors within 10%. Experimental average fatigue failure life of the specimens is 33,085 cycles, which is also close to the prediction with relative errors within 10%. This indicates the reliability and applicability of the established theoretical model and numerical method for predicting the failure of hat-shaped girder structures.

## 1. Introduction

Composite structures have the advantages of high specific strength, high specific stiffness and good designability. They have been widely used in the structural design of civil aircraft recently [1,2,3]. As a longitudinal member of the fuselage structure, the truss mainly bears the lateral force caused by the bending of the fuselage, and its compressive bearing capacity has an important influence on the stability and post-buckling bearing capacity of the integrally reinforced panel composed of the truss and the skin [4]. It is of great engineering significance to carry out failure analysis on the interface of reinforced panels, which can ensure the structural safety while exerting the advantages of material properties [5].

**Figure 1:**
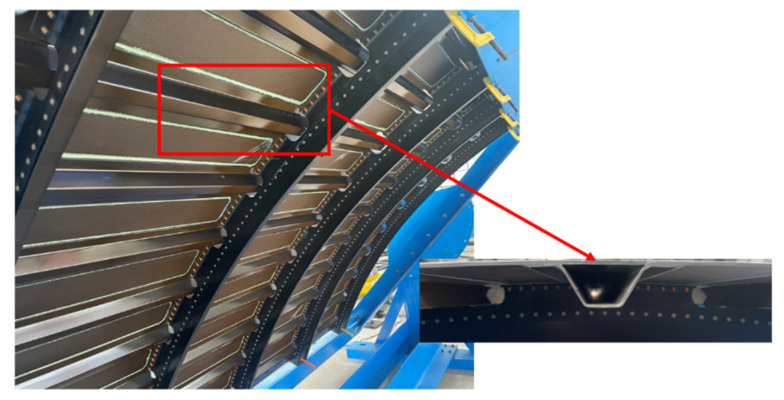
Hat-stringer-stiffened composite panels from the fuselage of an aircraft.

The composite girders and the skin are usually connected by co-bonding and co-curing, etc. to form reinforced panels. Different from the traditional “T-shaped”, “I-shaped” and other long girders, the composite hat-shaped long girders (as shown in Figure 1) have better bending stability and greater torsional stiffness, and are the most important part of the fuselage structure. With the development trend, 787 and A350XWB aircrafts have adopted a large number of long stringers with this section form in the composite fuselage panels [6].

In actual working conditions, the composite reinforced panel will bear not only the lateral force caused by the bending of fuselage, but also the radial pressure caused by the internal and external differential pressure during the take-off and landing of the aircraft. The latter case has a lack of investigation. The interface between the girder and the skin is the key position for the failure analysis of the panel. Delamination and debonding reduce the bearing capacity of the siding, and are common damage forms of composite reinforced panels. For the failure mechanism of debonding in composite hat-shaped truss reinforced panels, researchers have carried out certain experimental and numerical studies. Bertolin et al. [7] applied a four-point bending load to the hat-shaped long truss structure to simulate damage to the structure under local buckling load, used virtual crack closure technology (VCCT) to analyze the energy release rate at different crack initiation positions and found that mode I delamination occurs at the free end of the truss, delamination forms at the inner corners of the ribs and the skin is mode II slip-shear type. Sellitto et al. [8] studied the failure process of carbon fiber composite hat-reinforced notched wall panels under compressive loads through experiments and numerical simulations and revealed the progressive damage development of the fiber matrix caused by buckling and compressive loadings. They found that the mechanical and failure behavior was minimally influenced by the pre-existing damage. This influence can be observed when the buckling load is far exceeded. Sepe et al. [9] carried out static compression tests and finite element analysis of undamaged and damaged composite hat-shaped truss reinforced panels and used Hashin criterion to model the progressive damage of the structure. The mechanical properties of the structure under compressive load were evaluated and the residual strength of damaged structures was calculated. However, both debonding and buckling skin cannot be simulated. Riccio et al. [10] considered intralaminar and interlaminar damage to the hat-shaped truss reinforced panel structure, and studied the effect of the interaction between delamination and fiber-matrix damage in the large-notch damage region on the overall compressive performance of the structure. Using the cohesive zone model and Hashin criterion, combined with the material degradation law, a numerical method considering delamination and fiber-matrix damage evolution was established. It was found that the damage starts at the notch edges and propagates towards the panel edges leading to a net tension failure mode. Furthermore, Riccio et al. [11] conducted numerical and experimental studies on composite stiffened panels under low-velocity impact, and obtained load-time, load-displacement and energy-time curve considering two different levels of impact energy. Good agreement between experimental and numerical results was achieved. It shows that a model considering exclusively intra-laminar damage is adequate to describe the global behavior. Ji et al. [12] proposed a numerical mode combined with a progressive damage model and a cohesive zone model to study the effects of debonding defects on the post-buckling and failure behaviors of composite stiffened panels under uniaxial compression. It found that debonding defects can change post-buckling deformation modes and their development paths, and further weaken the ultimate failure strength of the stiffened panels. Vescovini et al. [13] proposed a semi-analytical method for evaluating the buckling response of hat-shaped girder stiffened panels under combined compressive and shear loadings based on the principle of minimum potential energy combined with the Ritz method. The comparison with the numerical results, presented for different panel lay-ups and aspect ratios, exhibits differences below 9% for the buckling load. Castro and Donadon [14] proposed a semi-analytical approach to evaluate the effect of debonding defects on the vibration and linear buckling behavior of T-stiffened composite panels. It shows that panels with high skin-to-stiffener ratio tend to be more skin-dominant and less sensitive to the defect size. Bisagni et al. [15] proposed a single-stringer specimen for assessing the post-buckling response of hat-stiffened composite panels and a shell-base finite element model was developed to predict the progressive damage process. Good agreement was found in the predicted collapse loads, differing from the experimental results by less than 6%. The effect of the embedded defect on the specimen strength was also accurately predicted, which reduces the collapse load by approximately 12%. The Bisagni group [16,17,18] also performed investigations on the fatigue behavior of post-buckled composite stiffened structures and a numerical method was proposed to evaluate the fatigue delamination growth in the structures. Mo et al. [19] conducted an experimental study on the buckling and post-buckling behaviors of hat-shaped long girders under axial compression. According to the experimental strain data and failure mode, the stability of the stiffened plate was analyzed. Based on the iSIGHT Built-in NLPQL algorithm, MATLAB code was written to calculate the optimization objective function and optimize the design of the hat-shaped truss. The results showed that the hat-stringer flat panel had a large load capacity after initial buckling. In addition, test fixtures play a significant role in experiments and accurate test results need good test fixtures.

From the above discussions, it can be seen that most of the existing research on composite hat-shaped truss reinforced panels focuses on the failure behavior under static loading, while there is a lack of research on the fatigue failure behavior. Especially for the fatigue properties under radial compression loading, there is still no report based on our best knowledge. In order to meet the damage tolerance design requirements of composite reinforced panels in engineering practice, it is necessary to further understand the failure behavior of the reinforced panel interface under fatigue loading. In this work, the radial compression properties of the hat-shaped long truss glued structure under static and fatigue loadings are studied. By simplifying the radial loading of the hat-shaped long truss structure to the bending form, four-point bending tests on hat-shaped truss specimens are performed under static and fatigue loadings. A novel theoretical model based on the fatigue damage theory has been proposed. In addition, a user-defined subroutine USDFLD is developed for the implementation of the proposed theoretical model. The failure mode and delamination growth process of the typical structure under fatigue loading are predicted, and the feasibility and accuracy of the numerical method in this work are verified by experimental results. This work can provide theoretical guidance and technical support for the damage tolerance design of composite hat-shaped truss stiffened panels.

The organization of the paper is as follows: In Section 2, a new theoretical model considering fatigue damage is proposed. Th specimen, test set-up and test method are introduced in Section 3. A finite element model is built in Section 4 followed by experimental results and numerical results presented in Section 5. Comparisons and discussions are also given. Finally, conclusions are drawn based on the above study.

## 2. Theoretical Model for Debonding Failure of Composite Materials Considering Fatigue Damage

The existing cohesive zone model in commercial software can only be used for the modelling of static delamination growth. In this section, the traditional cohesive zone model with bilinear constitutive law is introduced. Based on this, a novel theoretical model for fatigue delamination damage is proposed.

### 2.1. Cohesive Zone Model

Some previous methods based on fracture mechanics theory, such as J-integral theory, stiffness differential method and virtual crack closure technology, can only analyze structures containing cracks or delamination, but cannot simulate the initiation of cracks or delamination. These methods require predicting the initiation location of cracks or delamination, which is usually difficult for complex structures. The cohesive zone model emerging in recent years overcomes the shortcomings of the above methods and can simulate the initiation and growth of delamination at the same time.

When using the cohesive zone model, it is necessary to pre-arrange a layer of cohesive elements along the predicted crack or delamination path in the structures [20], and the cohesive layer should be connected with the continuous elements of the adjacent structures on both sides. Delamination can only occur along the preset path of cohesive elements, otherwise meshing with different paths is required to be built. The cohesive element is damaged and gradually loses stiffness and grows with the increase of external load. In this work, the bilinear constitutive law is used to carry out the finite element analysis of the failure in the hat-shaped truss-skin interface. The key to the finite element analysis of composite interface failure is to clarify the constitutive relationship and the interface failure criterion.

#### 2.1.1. Bilinear Constitutive Law

The cohesive zone model simplifies the interface characteristics to the interaction force at the microscopic level of the material. The constitutive law is very important for the macroscopic mechanical state of the cohesive region. The currently developed cohesive zone model generally assumes that the cohesive force is a function of the opening displacement of the crack surface [21,22]. The connection between the microscopic damage mechanism and the interface deformation is constructed through the cohesive element to simulate the interlayer interface response of the composite laminates. During the simulation process, the delamination will grow along the preset bonding path, damage will only occur in the cohesive elements, and the continuous elements on both sides will remain intact. Constitutive laws of cohesive elements mainly include bilinear, exponential, trapezoidal and multi-linear ones.

**Figure 2:**
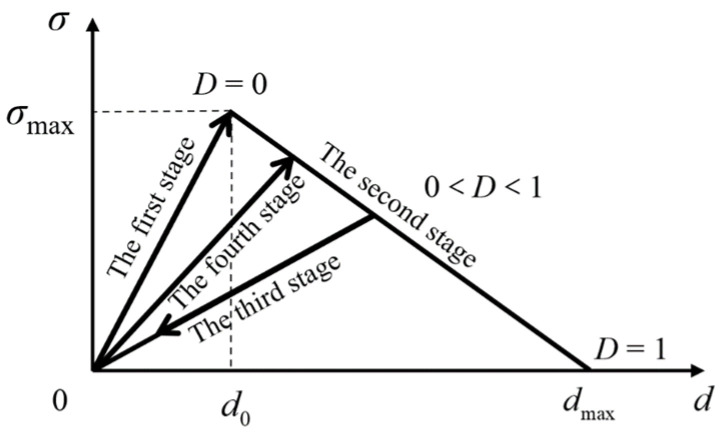
Sketch of the bilinear constitutive law.

The bilinear constitutive law is the simplest and most commonly used one in the cohesive zone model [23]. As shown in Figure 2, the damage state of the cohesive element is described by the damage factor *D*. Taking the mode I delamination for example, when the opening displacement 0 ≤ *d* ≤ *d*_0_, *D* = 0, the mechanical response of the cohesive element is in a non-damaged state in the linear elastic stage; when *d*_0_ < *d* ≤ *d*_max_, 0 < D ≤ 1, the cohesive element is in the damage state of the strain softening stage. When unloading, the stress-strain relationship will return to the origin along the arrow diagonal line as shown in the third stage of the figure. When *d* > *d*_max_, D > 1, the element is completely damaged and will lose its bearing capacity. In the delamination simulation, the upper and lower crack surfaces are completely separated and no longer interact.

Assuming that the loading and unloading processes are all linear processes, the constitutive relationship between the traction force *T* and the opening displacement *d* is as follows:

(1) $$T=\left\{\begin{array}{ll} \frac{\sigma_{\max}}{d_{o}}d & 0\leq d\leq d_{o}\\ \frac{\sigma_{\max}}{d_{o}-d_{\max}}d+\frac{\sigma_{\max}d_{\max}}{d_{\max}-d_{o}} & d_{o}<d\leq d_{\max}\\ \frac{\sigma_{\max}}{d_{o}}d\cdot \left(1-D\right) & un\;or\;\mathrm{re-loading}\\ 0 & d_{\max}\leq d \end{array}\right.$$

where *T* and *d* are the traction force and opening displacement in the current state, respectively, and *D* is the damage factor. Under the assumption that damage has been caused during the tensile process, the four stages in Figure 2 can represent loading, unloading and reloading, respectively, corresponding to the different traction-displacement relations in Equation (1). The first stage and the second stage are both static tensile stages, the third stage is the unloading stage, and the fourth stage is the reloading stage.

#### 2.1.2. Interface Failure Criteria

The interface failure criterion includes two stages of delamination initiation and delamination growth. Under pure mode I, mode II and mode III loadings, the initiation of delamination in composites can be determined by simply comparing the magnitude of the cohesive force component with the interfacial strength. When the cohesive force of the interface is equal to the interface strength in the corresponding delamination mode, the delamination initiation criterion can be expressed as Equation (2):

(2) $$\begin{array}{ll} \sigma_{I}^{}=\sigma_{I}^{0}, & \mathrm{mode}\;I\\ \sigma_{\mathrm{II}}=\sigma_{\mathrm{II}}^{0}, & \mathrm{mode}\;\mathrm{II}\\ \sigma_{\mathrm{III}}=\sigma_{\mathrm{III}}^{0}, & \mathrm{mode}\;\mathrm{III} \end{array}$$

where $\sigma_{I}^{0}$, $\sigma_{\mathrm{II}}^{0}$ and $\sigma_{\mathrm{III}}^{0}$ are mode I, mode II and mode III interfacial strengths, respectively. The interfacial strength corresponds to the traction peak of the traction-displacement relationship in the bilinear constitutive law. Because the interfacial strength characterizes the delamination onset during the formation stage of matrix micro cracks, it is usually difficult to directly measure its value through experiments. In the mixed loading mode, initial failure is likely to occur when the stress components are lower than the interfacial strength of a single mode. Therefore, a hybrid delamination initiation criterion considering the interaction of various stress components is proposed, mainly the quadratic stress failure criterion [24] and the quadratic strain failure criterion, as Equations (3) and (4), respectively.

(3) $${\left\{\frac{\langle \sigma_{I}\rangle }{\sigma_{I}^{0}}\right\}}^{2}+{\left\{\frac{\sigma_{\mathrm{II}}}{\sigma_{\mathrm{II}}^{0}}\right\}}^{2}+{\left\{\frac{\sigma_{\mathrm{III}}}{\sigma_{\mathrm{III}}^{0}}\right\}}^{2}=1$$

(4) $${\left\{\frac{\langle \varepsilon_{I}\rangle }{\varepsilon_{I}^{0}}\right\}}^{2}+{\left\{\frac{\varepsilon_{\mathrm{II}}}{\varepsilon_{\mathrm{II}}^{0}}\right\}}^{2}+{\left\{\frac{\varepsilon_{\mathrm{III}}}{\varepsilon_{\mathrm{III}}^{0}}\right\}}^{2}=1$$

The delamination growth of the composite material can be judged by comparing the strain energy release rate and the fracture toughness in the corresponding delamination mode. When the strain energy release rate reaches the fracture toughness in the corresponding mode, delamination growth occurs, as shown in Equation (5), otherwise there is no growth.

(5) $$\begin{array}{ll} G_{I}=G_{\mathrm{IC}}, & \mathrm{mode}\;I\\ G_{\mathrm{II}}=G_{\mathrm{IIC}}, & \mathrm{mode}\;\mathrm{II}\\ G_{\mathrm{III}}=G_{\mathrm{IIIC}}, & \mathrm{mode}\;\mathrm{III} \end{array}$$

where *G*_IC_, *G*_IIC_ and *G*_IIIC_ stand for the mode I, mode II and mode III fracture toughness, respectively. The value of pure mode fracture toughness is equal to the area under the traction-displacement curve as shown in Figure 2. The delamination growth under the mixed mode loading may occur before the strain energy release rate component in each single mode reaches the fracture toughness value, so the delamination growth criterion proposed for the single mode is no longer applicable. Commonly used delamination growth criteria in mixed mode include power law criterion and B-K criterion [25,26], which are as Equations (6) and (7), respectively:

Wu and Reuter [27] proposed the power law criterion for mixed mode I/II delamination:

(6) $${\left(\frac{G_{I}}{G_{\mathrm{IC}}}\right)}^{\alpha }+{\left(\frac{G_{\mathrm{II}}}{G_{\mathrm{IIC}}}\right)}^{\alpha }=1$$

where *G*_I_ and *G*_II_ represent the strain energy release rates of the mode I and mode II delamination, respectively. However, the power law criterion cannot accurately capture the dependence of the fracture toughness of epoxy composites on the loading mixture ratio. To overcome this difficulty, Benzeggagh and Kenane [28] proposed the B-K criterion. This criterion is represented by the mode I and mode II fracture toughness and the parameter *η* based on the test results of MMB under different loading and mixing ratios:

(7) $$G_{\mathrm{IC}}+\left(G_{\mathrm{IIC}}-G_{\mathrm{IC}}\right){\left(\frac{G_{\mathrm{II}}}{G_{T}}\right)}^{\eta }=G_{C},\;G_{T}=G_{I}+G_{\mathrm{II}}$$

### 2.2. Bilinear Cohesive Zone Model with Fatigue Damage

The above traditional bilinear cohesive zone model does not take into account the damage accumulation process. Therefore, when studying the fatigue damage failure behavior of the hat-shaped truss interface, the traditional cohesive zone model should be combined with damage accumulation to develop a new one considering the fatigue damage.

During cyclic loading, the material will occur fatigue damage until failure. From the macroscopic point of view, fatigue damage often manifests as the degradation of material stiffness. In the cohesive zone model considering fatigue damage, the degradation of material stiffness can be characterized by the damage factor *D*. The interfacial damage can usually be divided into two types [24]: static damage caused by excessive one-time loading and fatigue damage caused by cyclic loading.

According to the damage theory of Roe and Siegmund [29], in the case of cyclic loading, the cohesive zone model will undergo stress degradation, the relationship between the maximum stress *σ*_max_, *τ*_max_ and the damage factor *D* is:

(8) $$\sigma_{\max}=\sigma_{\max,0}\left(1-D\right),\;\tau_{\max}=\tau_{\max,0}\left(1-D\right)$$

where *σ*_max,0_ and *τ*_max,0_ are the initial maximum normal and tangential stresses, respectively. When the cumulative damage factor reaches 1, it means that the material has undergone complete fatigue failure and crack propagates. *D* can be divided into two parts: *D_c_* for the fatigue damage and *D_m_* for the static damage. The calculation criteria for fatigue damage are as follows:

(9) $${\dot{D}}_{c}=\frac{\left|\Delta \dot{\bar{u}}\right|}{\delta_{\Sigma }}\left(\frac{{\dot{T}}_{n}}{\sigma_{\max}}-f_{0}\right)H\left(\Delta \bar{u}-\delta_{0}\right),\;D_{c}\geq 0$$

$\Delta \dot{\bar{u}}$ Displacement increment,
${\dot{T}}_{n}$ Stress increment,
$\sigma_{\max}$ Maximum stress of the cohesive element,
*H* Heaviside function,
$\Delta \bar{u}$ Sum of the cumulative displacement increment,
$\delta_{0}$ Characteristic displacement of the cohesive zone model.

For the bilinear cohesive zone model of this paper, the characteristic displacement $\delta_{0}$ is *d*_0_. $\delta_{\sum }$ and *f*_0_ are parameters of fatigue damage. $\delta_{\sum }$ is used as the damage threshold, acts as a scaling factor for calculating the damage due to effective displacement increments and its value can be several times of $\delta_{0}$. *f*_0_ is used as a threshold factor, its value is between 0 and 1 and it represents the ratio between the stress amplitude $\sigma_{f}$ and the initial maximum stress $\sigma_{max,0}$ of the cohesive zone model.

Under static loading, if the difference between the maximum value of the current displacement $\Delta {\bar{u}}_{t}$ and the maximum value of the displacement $\Delta {\bar{u}}_{t-\Delta t}$ at the previous moment is greater than the characteristic displacement $\delta_{0}$, the static damage ${\dot{D}}_{m}$ is calculated as follows:

(10) $${\dot{D}}_{m}=\frac{\max\left(\Delta {\bar{u}}_{t}\right)-\max\left(\Delta {\bar{u}}_{t-\Delta t}\right)}{4\delta_{0}},\max\left(\Delta {\bar{u}}_{t-\Delta t}\right)>\delta_{0}$$

The total damage value can be obtained by accumulating fatigue damage and static damage *D*:

(11) $$D=\int \max\left({\dot{D}}_{c},{\dot{D}}_{m}\right)dt$$

Considering that the strength of the interface is different in the normal direction and the tangential direction, different cohesive parameters should be used to distinguish it in the tangential direction and the normal direction. However, both of them are related to the damage degree of the material. Therefore, this work adopts the overall damage judgment method to evaluate the damage behavior of the material, and uses the displacement value and stress value at the interface to calculate the material damage. The specific formula is as follows:

(12) $${\dot{\delta }}_{n}=\sqrt{\delta_{normal}^{2}+\delta_{shear}^{2}}$$

(13) $${\dot{T}}_{n}=\sqrt{T_{normal}^{2}+T_{shear}^{2}}$$

*δ_normal_*  normal displacement
*T_normal_*  normal stress
*δ_shear_*  shear displacement
*T_shear_*  shear stress

**Figure 3:**
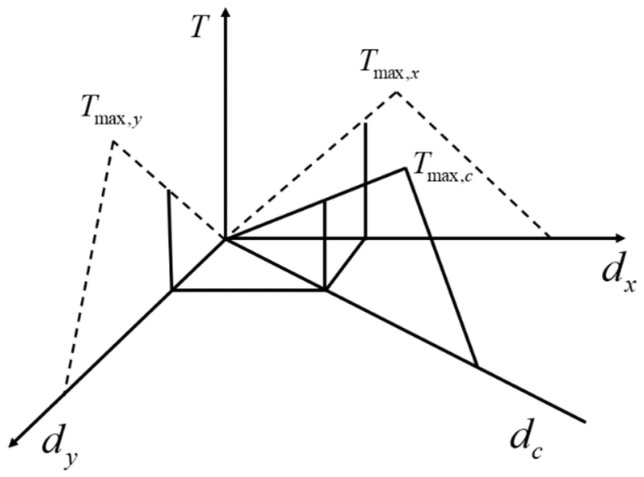
Schematic diagram of right-angle coupling between the tangent planes.

However, in the finite element analysis, variables in three directions need to be provided in the Cartesian coordinate system. Considering the isotropy of the material on the tangent plane, the method of coupling calculation is adopted. The displacement and stress in the upper two directions are coupled to obtain the actual displacement value and direction on the plane (as shown in Figure 3), and then the cohesive criterion is used for calculation in the current direction.

The specific coupling method can refer to Equation (14). After the calculation of the damage, the coupling result can be decomposed into the original direction (that is, the X and Y directions) by Equations (15) and (16) for the next finite element calculation.

(14) $$d_{c}=\sqrt{d_{x}^{2}+d_{y}^{2}}$$

(15) $$T_{x}=d_{x}\frac{T_{c}}{d_{c}}$$

(16) $$T_{y}=d_{y}\frac{T_{c}}{d_{c}}$$

where *d_c_* and *T_c_* respectively represent the displacement value after coupling and the traction force is calculated according to the cohesive criterion.

## 3. Materials and Methods

### 3.1. Specimen Introduction

**Figure 4:**
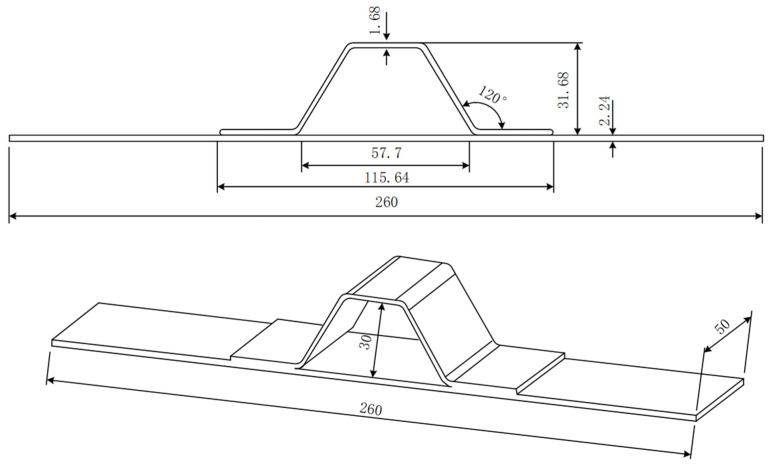
The configuration and geometry of specimen. (Unit: mm).

The hat-shaped truss specimen is composed of a skin and a long truss. The long truss and the skin are formed by a co-bonding process with adhesive films. The mechanical properties of the composite material are shown in Table 1, the dimensions of the test specimens are shown in Figure 4, the thickness of one single layer of the prepreg after curing is 0.187 mm, and the layer information of each part is shown in Table 2.

### 3.2. Test Set-Up and Method

**Figure 5:**
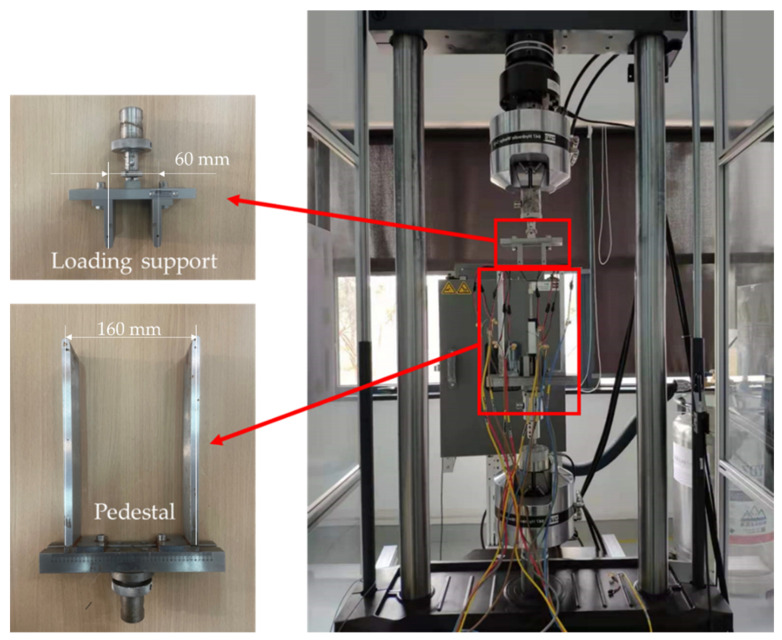
Set-up for the four-point bending test.

**Figure 6:**
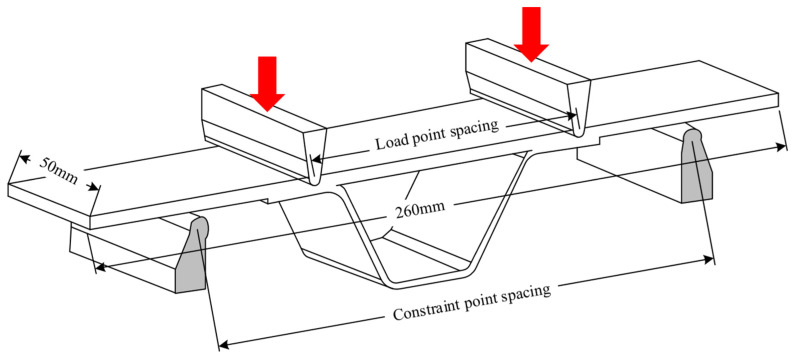
Schematic diagram of the four-point bending loading.

The four-point bending test set-up is shown in Figure 5, and the loading schematic diagram is shown in Figure 6. As shown in Figure 6, the distance between the loading point and the restraint point is 60 mm and 160 mm respectively, and the loading point is located in the R zone of the hat-shaped long stringer. The static and fatigue tests are conducted on an INSTRON testing machine and an MTS hydraulic testing machine, respectively. All tests are carried out in a standard atmospheric environment with a temperature of 23 ± 2 °C and a humidity of 50 ± 10%. Before four-point bending tests, nondestructive checking on the specimens is carried out using special nondestructive testing equipment (GE USN60). Only specimens without defects are used. Four-point bending static and fatigue tests are performed referring to the standard ASTM D7264 [30]. The failure fracture surfaces of the specimen are observed by the microscope ZEISS Axiocam 506 (Jena, Germany).

The four-point bending static test of the composite hat-shaped girders adopts the displacement loading mode. The displacement load is gradually applied until the failure of the tested specimen with a loading rate of 1 mm/min. The loading direction of the load is parallel to the axial direction of the long side of the tested specimen. The loading center line passes through the pressure center of the section of the specimen. It is necessary to record the original state and the state of the R area and the co-bonding interface area of the specimen before and after the test, and observe whether the tested specimen is delaminated and debonded.

Under cyclical loading, the structure usually encounters delamination at the R zone after certain cycles, as shown in Section 5. The four-point bending fatigue tests in this study can well reproduce the damage behavior of the structures. Because there is still no international standard for fatigue tests of the hat-shaped girders [31], the standard ASTM D7264 for static tests is referred in terms of the specimen form, loading method and data reduction method. Only the incremental displacement loading is changed to cyclic fatigue loading. The initial load applied in the fatigue test is taken as a percentage based on the average value of the initial debonding load, which can be determined from the static test. The setting of the loading point and support point is the same as that of the static test. The fatigue test procedures are as follows: The spacing between support bases in the four-point bending fixture is set as 160 mm and the spacing between loading supports is set as 60 mm. The upper and lower supports and specimen are then fixed in the test set-up. The displacement corresponding to 90% of the average value of the initial debonding load in static tests is taken as the initial loading displacement of the fatigue test [31,32]. The stress ratio is 0.06 and the loading frequency is 1.5 Hz. Visual inspection on the specimen is performed every 10,000 cycles. When the feedback force value of the testing machine changes significantly, the test will be stopped and the specimen will be removed from the test device. The failure mode and failure area of the tested specimen should be checked and recorded.

## 4. Finite Element Model

**Figure 7:**
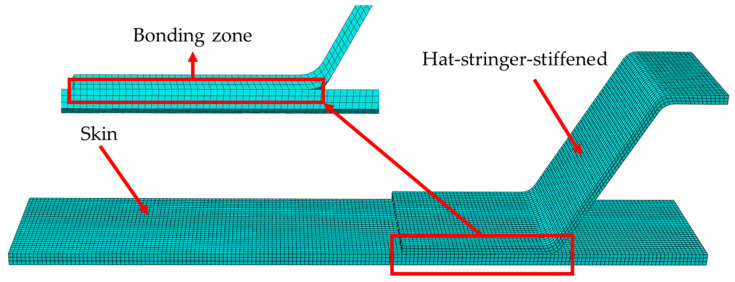
3D finite element model of the four-point bending test.

Using the commercial finite element software ABAQUS^®^ (version 6.14, Dassault Systemes, Paris, France), a three-dimensional finite element model is established for the four-point bending of the hat-shaped truss-skin in the lower wall of the composite fuselage. Considering that the structure of the tested specimen and the loading form are completely symmetrical, a quarter of the specimen is modelled as shown in Figure 7. Both the girder and the skin use SC8R continuous shell elements, and the girder and the skin are divided into 9 layers and 12 layers respectively, and the thickness of the single layer is 0.187 mm.

By monitoring the change of the overall stiffness of the structure with different mesh refinement degrees, the mesh convergence analysis of the established finite element model is carried out. When the mesh size is 0.75 mm, the finite element results can be sufficiently accurate. The co-bonding interface between the hat-shaped stringer and the skin is simulated by inserting the COH3D8 cohesive element with zero thickness, and the element deletion is defined to simulate the debonding and delamination behavior of the co-bonding area. The interfacial parameters of the co-bonding interface are shown in Table 3. The 3D discrete rigid body is used to model the loading support and the support base. The surface–surface contact between the upper and lower surfaces of the skin and the rigid body is defined. The “hard contact” is set to avoid penetration during the analysis process. The support base is fully clamped. The loading support is applied with a displacement loading. In order to ensure that the analysis and calculation process has good convergence, a viscosity coefficient value of 10^−5^ is set [33]. The displacement and support–reaction force data at the loading point are extracted during post-processing to obtain the numerical load-displacement curves.

## 5. Results and Discussion

### 5.1. Experimental and Numerical Results of Static Tests

#### 5.1.1. Experimental Results

The typical failure modes of the composite reinforced panels mainly include: the debonding failure between the adhesive layer and the glued parts, the failure of the glued parts, the fracture failure of the glue layer and mixed failure of the above three kinds. Different failure modes correspond to different mechanisms, such as the debonding failure between the adhesive layer and the glued part, which may be caused by improper surface treatment of the glued part during the bonding process, and the fracture failure of the adhesive layer may be caused by poor mechanical properties of the adhesive itself. For good design and craftsmanship, the bond strength should be greater than the strength of the part to be glued, that is, the debonding failure mode of the specimen should be the failure of the part to be glued.

**Figure 8:**
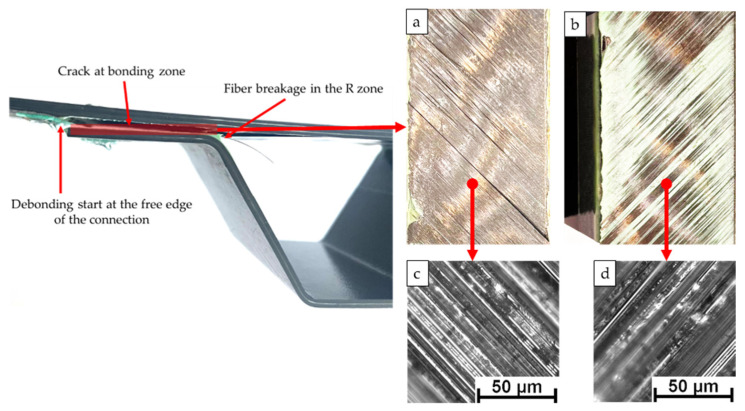
(**a**–**d**) Failure modes and fracture surfaces of the static tests.

The damage state at the stringer-skin when the crack propagates is shown in Figure 8. The initial debonding of the specimen occurs at the free edge of the connection between the truss flange and the skin. The debonding failure occurs in the glued part, including delamination and fiber fracture of the skin, which can be seen from the fracture surfaces of the skin and the girder. This indicates that, at least in the four-point bending test, the bonding quality between the truss flange and the skin meets the static strength requirements. As the load increases, the delamination failure area also increases, and finally one side edge of the stringer is separated from the skin. When the structure fails completely, a large area of debonding occurs at the interface.

**Figure 9:**
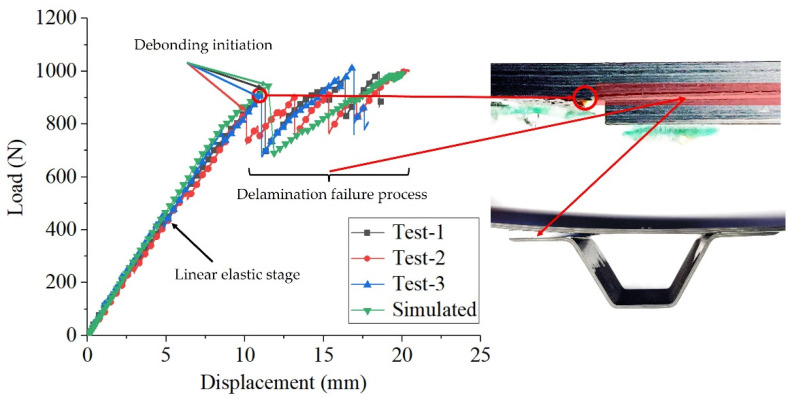
Load-displacement curves of the static tests and comparison with numerical result.

Figure 9 shows the load-displacement curves of the four-point bending static test from the three same specimens. It can be seen that the load-displacement curves of the tested specimens have good consistency during the linear elastic stage and the debonding failure process, indicating that the static tests carried out have less dispersion and the results are reliable. When the displacement load increases to around 10.8 mm, the delamination begins to propagate and the local material failure causes the load to drop. After the first drop of the load, all the tested specimens have the ability to bear larger loads, which shows that the structure can continue to bear the load after the initial debonding at the free edge of the connection area between the girder flange and the skin. The average value of the maximum load from all specimens is 897.3 N. A large area of debonding occurs at the interface between the long stringer edge and the skin, and the damage spreads rapidly on the skin, resulting in the eventual complete failure of the skin. Table 4 concludes the initial debonding load and corresponding initial debonding displacement (the displacement of the loading point at the time of debonding) for the three tested specimens.

#### 5.1.2. Numerical Results

**Figure 10:**
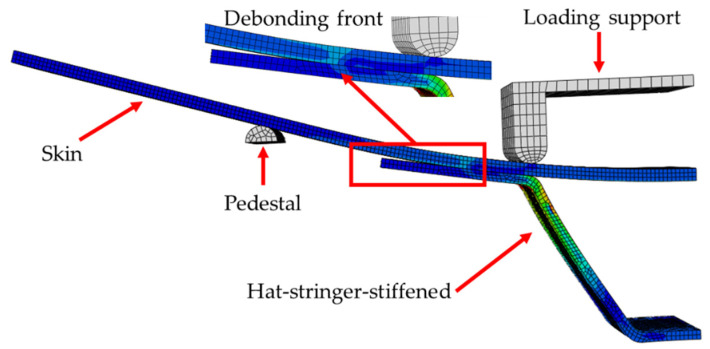
Numerical results of the static four-point bending tests.

The finite element analysis mainly examines the ability of the cohesive element to simulate the fracture failure of the adhesive layer. The failure criterion is shown in Section 2. In the numerical study of static test, a displacement loading of 20 mm is set for the loading support in the finite element model. The predicted load-displacement response and the failure pattern are compared with experimental results. Numerical results show that when the displacement loading reaches 11.49 mm, the initial debonding of the interface occurs, as shown in Figure 10. This is consistent with the test results. Figure 9 shows the comparison between the numerical load-displacement curve and the test ones. It can be seen that the predictions are in good agreement with the test results at the linear elastic stage and the debonding failure stage. The initial debonding load and corresponding displacement obtained from numerical results are 944.19 N and 11.49 mm, respectively. Relative errors with the average value of initial debonding load (897.3 N) and the average value of initial debonding displacement (10.8 mm) from the static tests are within 10%. It is verified that the established 3D finite element model of the hat-shaped girders-skin four-point bending is reasonable and effective, and its applicability in predicting the interface debonding failure behavior of hat-shaped girder structures is demonstrated.

### 5.2. Experimental and Numerical Results of Fatigue Tests

#### 5.2.1. Experimental Results

From the static test results, it can be known that the average value of the initial debonding displacement is 10.8 mm, so the initial displacement loading of the fatigue test is 9.72 mm. Fatigue tests are carried out on seven specimens, and Table 5 summarizes the cyclic loading times and the interface failures of all specimens. It can be concluded that the average fatigue failure life of the reinforced panel interface of the hat-shaped truss is 33,085 cycles, and the fatigue damage mostly starts in the R zone of the hat-shaped truss and the free edge of the connection between the truss flange and the skin. The main failure modes of the configuration are the delamination of the skin of the glued part and the debonding of the girder flange from the skin. The fatigue failure life of the specimens is obviously different, reflecting the instability of the fatigue life, which may be caused by the imperfect process of the structure in the manufacturing process.

#### 5.2.2. Numerical Results

**Figure 11:**
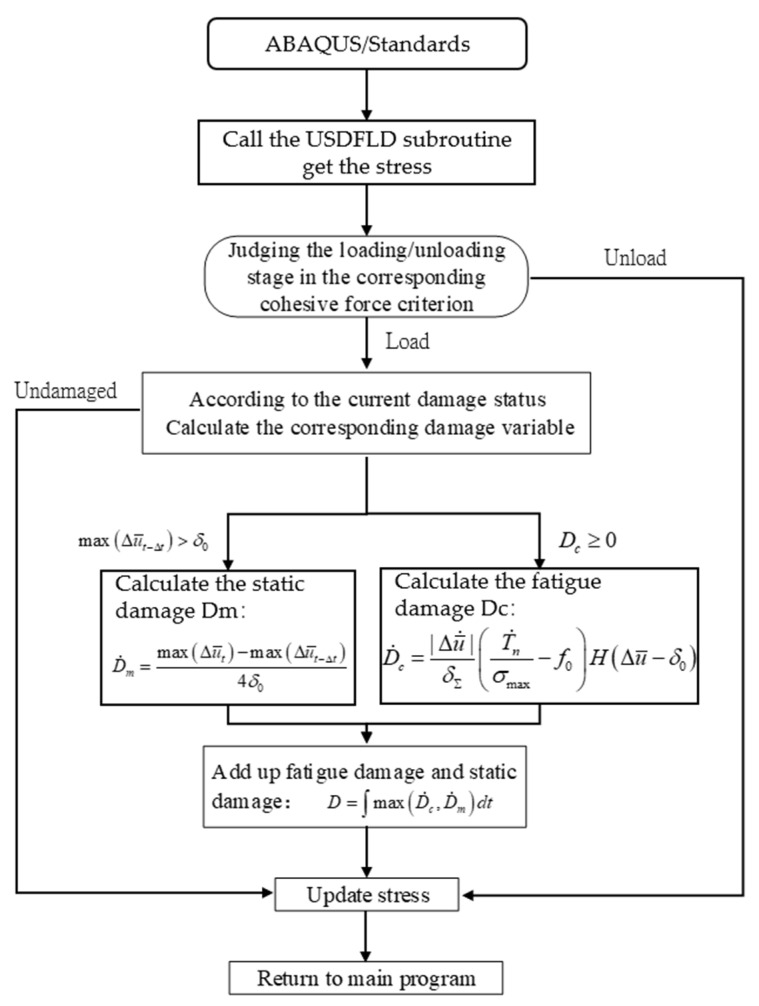
Flowchart for the implementation of damage calculation criteria for predicting the fatigue damage.

In the fatigue model, the loading form in the three-dimensional model of the hat-shaped truss-skin four-point bending static test is changed to the cyclic load with an amplitude of 9.72 mm. The maximum number of loading cycles is set to 100,000. To realize the prediction of fatigue damage, a subroutine USDFLD is developed and used in the ABAQUS finite element software. The cohesive zone model is embedded, and the damage calculation criterion is used to determine whether there is element damage at the truss-skin interface. The most critical part is the implementation of the fatigue damage criterion in the subroutine USDFLD. The flowchart of this part is shown in Figure 11.

**Figure 12:**
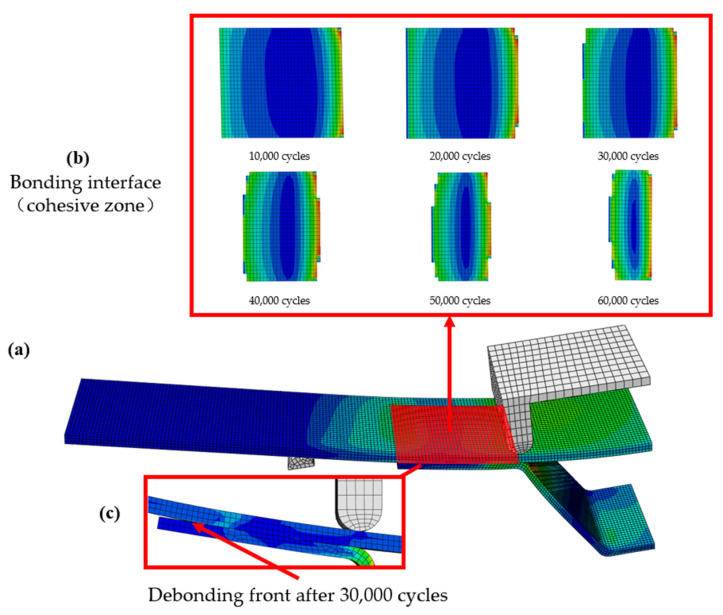
(**a**–**c**) The evolution process of interface fatigue damage in the four-point bending test.

The damage evolution at the interface can be characterized by examining the damage at the interface in different load steps. The damage and its location and type after certain cycles of fatigue loading is observed to understand the progressive damage behavior and compared with numerical analysis. The damage cloud diagram at the girder-skin adhesive interface is shown in Figure 12. Figure 12b shows the fatigue damage at the adhesive interface after 10,000, 20,000, 30,000, 40,000, 50,000 and 60,000 cycles of fatigue loading. After about 20,000 cycles, some damages in the R zone can be observed. After about 30,000 cycles, delamination occurs in the R zone as shown in Figure 12c, which is close to the average loading times of 33,085 times from the fatigue test results, considering the test scatter of composite structures. This indicates the reliability and applicability of the cohesive zone model considering fatigue damage in predicting the fatigue life of hat-shaped long truss structures. At the same time, it can also be noticed that the accumulation of damage often starts from both sides of the bonding interface, and gradually propagates to the middle of the interface with the increase of the number of cycles, until the final debonding failure after about 60,000 cycles. This indicates that the connection between the truss and the skin and the R zone are the weakest parts, where damage is most likely to be caused. Using the proposed cohesive zone model considering fatigue damage to predict the fatigue life of the hat-shaped truss-skin interface not only has a certain adaptability, but also can characterize the damage evolution process of the interface to a certain extent.

## 6. Conclusions

Based on fatigue damage theory, this study establishes a cohesive zone model considering fatigue damage, and conducts experimental research of hat-shaped truss structures under static and fatigue four-point bending loads. In addition, through the development of the Abaqus subroutine USDFLD, the numerical analysis of the interface failure of the structure is realized. Compared with the traditional life prediction theory, the cohesive zone model can provide better understanding of the damage evolution process. These works can provide theoretical guidance and technical support for the damage tolerance design of composite hat-shaped truss reinforced wall panels and have certain engineering application prospects. The main conclusions are as follows:

(a) Based on the bilinear constitutive law of the cohesive zone model and the interface failure criterion, a cohesive zone model considering fatigue damage is established, which effectively combines the traditional cohesive zone model with the damage accumulation.
(b) Specimen information is introduced. Test set-up is designed for four-point bending static and fatigue tests of the composite hat-shaped girders, which are performed referring to the standard ASTM D7264.
(c) A 3D finite element model is established for the four-point bending of hat-shaped long truss glued structures. Only a quarter of the specimen is modelled because the structure of the tested specimen and the loading form are completely symmetrical. The co-bonding interface between the hat-shaped stringer and the skin is simulated by inserting COH3D8 cohesive elements with zero thickness.
(d) The main failure modes of the static tests of the hat-shaped truss structure are the delamination of the skin and the fiber fracture of the glued parts. The initial debonding load and corresponding displacement obtained from numerical results are 944.19 N and 11.49 mm, respectively. Relative errors with the average value of initial debonding load (897.3 N) and the average value of initial debonding displacement (10.8 mm) from the static tests are within 10%.
(e) The results of the fatigue tests show that the R zone of the hat-shaped truss and the free edge of the truss flange and the skin connected are the weakest parts of the structure that are most likely to cause fatigue damage. The average fatigue failure life of the studied interface is 33,085 cycles, which is also close to the prediction with relative errors within 10%. The fatigue failure mode is the delamination of the skin and the debonding between the girder flange and the skin. In addition, the accumulation of fatigue damage often starts from both sides of the bonding interface, and gradually propagates to the middle of the interface with the increase of the number of cycles, until the final debonding failure.
(f) The proposed theoretical model can reasonably describe the fatigue failure of the glued interface. The numerical method proposed in this study can provide accurate prediction for the static failure behavior and fatigue life of hat-shaped girder structures.

## Figures and Tables

**Table 1:** Basic material parameters.

Young Modulus and Poisson Ratio	Value
*E*_11_/GPa	163.5
*E*_22_/GPa	9.00
*ν*_12_	0.32
*G*_12_/GPa	4.14
*G*_23_/GPa	3.08

**Table 2:** Layer information of the specimen.

Structure	Stacking Sequence	Layer Number	Nominal Thickness/mm
Skin	[45/−45/−45/90/45/0]	12	2.244
Truss	[45/0/0/−45/90/−45/0/0/45]	9	1.683

**Table 3:** Interfacial parameters of cohesive elements.

Interfacial Parameters	Value
$\mathrm{Nominal}\;\mathrm{tensile}\;\mathrm{strength}\;\sigma_{I}^{0}$/MPa	20
$\mathrm{Longitudinal}\;\mathrm{shear}\;\mathrm{strength}\;\sigma_{\mathrm{II}}^{0}$/MPa	30
$\mathrm{Transverse}\;\mathrm{shear}\;\mathrm{strength}\;\sigma_{\mathrm{III}}^{0}$/MPa	30
Normal tensile fracture energy *G*_IC_/(N·mm^−1^)	0.2
Longitudinal shear fracture energy *G*_IIC_/(N·mm^−1^)	2
Transverse shear fracture energy *G*_IIIC_/(N·mm^−1^)	2

**Table 4:** The initial debonding load and displacement of the tested specimens.

Specimen No.	Initial Debonding Load (N)	Initial Debonding Displacement (mm)
1#	935.6	11.3
2#	846.8	10.1
3#	909.5	11.1
Average	897.3	10.8
CoV	4.15%	4.86%

**Table 5:** Fatigue failure life and main failure mode of the test specimens.

Test Specimen No.	Cycles	Main Failure Mode
F-1*#*	31,990	Debonding at the R zone
F-2*#*	16,738	Delamination at the R zone
F-3*#*	23,361	Delamination at the R zone
F-4*#*	46,001	Debonding at the free edge
F-5*#*	28,460	Intralaminar delamination
F-6*#*	48,327	Delamination at the R zone
F-7*#*	36,717	Delamination at the R zone
Average	33,085	

## Data Availability

Not applicable.
